# Evaluation of cardiac diagnostic tests findings based on pro-BNP levels in COVID-19 pregnant patients

**DOI:** 10.1186/s12879-023-08764-1

**Published:** 2023-11-13

**Authors:** Mahdi Mazandarani, Rahmat Sharififar, Narges Lashkarbolouk, Somayeh Ghorbani

**Affiliations:** 1 Endocrinology and Metabolism Research Center, Endocrinology and Metabolism Clinical Sciences Institute, Tehran University of Medical Sciences, Tehran, Iran; 2 Golestan University of Medical Sciences, Gorgan, Iran; 3 Infectious Diseases Research Center, Golestan University of Medical Sciences, Gorgan, Iran; 4 Assistant Professor of Biostatistics, Golestan University of Medical Sciences, Gorgan, Iran

**Keywords:** COVID-19, Pro-BNP, Pregnancy, Cardiovascular system

## Abstract

**Background:**

Pro–b-type natriuretic peptide (Pro-BNP) is an inflammatory marker that indicates cardiac damage and inflammation. The elevation of this marker in COVID-19 patients can be used as a predictive factor in the prognosis of these patients.

**Method:**

Our cross-sectional study investigated the evaluation of cardiac diagnostic test findings based on pro-BNP levels in pregnant COVID-19 patients in Sayyad Shirazi Hospital, Gorgan, Iran, in 2020–2022. A hundred and ten pregnant patients diagnosed with COVID-19 infection were evaluated for cardiac diagnostic tests (electrocardiogram (ECG) and echocardiography (Echo)) and pro-BNP levels. Data were analyzed using SPSS 25 software. Chi-square and Student's t-test will be used to test and compare the relationship between variables and compare them. A *P*-value less than 0.05 is considered statistically significant. The chi-square test was used to compare the ratio of qualitative variables among the groups if the presuppositions of chi-square distribution were established. Otherwise, Fisher's exact test was used.

**Result:**

The mean age of participants were 31.06 ± 5.533 years and 49.1% of patients had pro-BNP levels above the cut-off value for predicting an adverse outcome of COVID-19. The mean ± standard deviation of pro-BNP levels in the low group was 46.125 ± 17.523 pg/mL and in the high group was 878.814 ± 1038.060 pg/mL. This study revealed that patients with higher pro-BNP plasma levels had a significant relation between, myocardial infarction (MI), pericardial effusion (PE), urgent Caesarean section (C/S), and mortality. In addition, no significant relation between gravid, trimester, vaccination, arrhythmia, heart block, and valves diseases with high pro-BNP levels was found.

**Conclusion:**

The current research showed that pro-BNP levels can be used as a diagnostic and valuable prognostic tool in pregnant women to diagnose cardiac complications by using ECG and Echo.

## Introduction

In 2019, the severe acute respiratory syndrome coronavirus 2 (SARS-CoV-2) (COVID-19) spread rapidly, resulting in severe acute respiratory syndrome. This disease is transmitted by airborne and the incubation period was one to fourteen days (an average of 3 to 7 days). COVID-19 can affect many organs, especially the cardiovascular system, and mortality increases due to comorbidities such as cardiovascular disease, hypertension, diabetes, chronic lung disease, and cancer [1–4].

Cardiovascular complications have become a significant cause of deterioration and mortality in about 8–12% of all patients during the COVID-19 pandemic. Patients with COVID-19 may have a direct cardiac injury or injury associated with systemic inflammation, hemodynamic instability, exaggerated cytokine response, and multi-organ failure. Cardiac biomarkers reported in cardiac damage and arrhythmia are troponin, N-terminal pro-B-type natriuretic peptide (pro-BNP), and myocardial creatine kinase band, which were reported significantly higher in severe COVID-19 patients. Further, the patients with a history of cardiovascular disease, COVID-19 increased cardiac markers associated with myocardial injury and arrhythmia [2–7].

Pro-BNP is an inflammatory cardiac marker, and the increase of this plasma marker reflects cardiac insufficiency and inflammatory condition such as COVID-19. Various pathophysiological pathways were responsible for raising the pro-BNP level after COVID-19 infection, and this marker could be used as a predictor in the prognosis and monitoring of COVID-19 patients. Studies have mentioned that high levels of this marker are associated with arrhythmias, cardiac block, myocardial ischemia (MI), diastolic dysfunction, and cardiac remodeling. Also, elevated pro-BNP can predict adverse clinical events, severe disease, and high mortality during the COVID-19 pandemic [2, 3, 6–9].

Cardiovascular complications during the epidemic can be investigated using non-invasive imaging methods such as electrocardiogram and echocardiography. ECG shows changes in acute myocardial ischemia, atrial tachycardia, ventricular tachycardia, and cardiac conduction disturbances (cardiac block). Echocardiography frequently demonstrates subclinical left ventricular diastolic impairment (EF), pericardial effusion, arterial thrombotic events, venous thromboembolic disease, and valvar dysfunction [5, 7–12].

Pregnancy with different physiological effects on the cardiovascular system puts this system at high risk of adverse effects during labor and early postpartum. In addition, COVID-19 infection has been associated with myocardial injury, which may increase the adverse impact on pregnant patients. The risk of severe infection was increased in pregnant women who are positive for COVID-19 compared with non-pregnant patients. In addition, an increase in the number of ICU admissions, invasive ventilation, and extracorporeal membrane oxygenation requirements and mortality was reported to be higher. Medications that can treat COVID-19 in pregnant women and pose a low or negligible risk in pregnancy are difficult to find. In addition, pregnant women cannot directly participate in clinical studies, which raises suspicions [3–11].

Cardiovascular complications remain a major challenge for pregnant patients' management and therapeutic interventions during the pandemic. Therefore, using pro-BNP as a diagnostic and prognostic marker to monitor cardiac function and cardiovascular dysfunction in the early stages of COVID-19 is useful. Few Studies focused on the relation between pro-BNP and cardiac diagnostic tests findings have not been investigated [6–8, 11–13].

Thus, this study aims to evaluate cardiac diagnostic test findings based on pro-BNP levels in COVID-19-positive pregnant women.

## Material and methods

### Study design and participant's selection

This cross-sectional study was performed on pregnant patients diagnosed with covid-19 and hospitalized at Sayad Shirazi Hospital in Gorgan. The inclusion criteria were pregnant women without a history of cardiac disease and confirmation of infection with the SARS-CoV-2 fluorescent reverse transcription quantitative real-time polymerase chain reaction (RT-qPCR) method. Patients with a history of cardiac surgery and disease and those who did not have ECG, Echo, and pro-BNP tests were excluded from the study.

### Data collection

Patients’ characteristics include demographic data including demographics and clinical characteristics were collected through a patient record system. Based on the variables checklist, data was collected from laboratory results and electrocardiography (ECG) and echocardiography (Echo) reports.

According to the World Health Organization's guidance on COVID-19, the participants were all pregnant patients diagnosed by infection specialists. Patients were screened for cardiac diagnostic tests (electrocardiogram and echocardiogram) and tested for pro-BNP levels during admission. The total number of pregnant patients was enrolled in the study was 110.

The use of the PCR method to diagnose infectious diseases has increased due to its high sensitivity and specificity. So, the COVID-19 RT-qPCR for confirming the diagnosis of COVID-19 was used in this study [14]. Patients were divided into high and low levels of pro-BNP, and the cut-off value for this classification was the average level of 88.64 pg/mL, according to Gao's study [7].

### Statistical analysis

Data were analyzed using Statistical Package for Social Sciences version 25.0 (SPSS Inc, Chicago, IL.), and Chi-square and Student's t-test will be used to test and compare the relationship between variables and compare them. A *P*-value less than 0.05 is considered statistically significant. The chi-square test was used to compare the ratio of qualitative variables among the groups if the presuppositions of chi-square distribution were established. Otherwise, Fisher's exact test was used. The outcomes are expressed as mean ± standard deviation or standard error.

## Result

After implementing inclusion and exclusion criteria, a hundred-ten pregnant patients were enrolled to the study. Participants were 14–44 years old, with a mean age of 31.06 ± 5.53 years. Our study reported no statistically significant differences between the age of the patients in the two groups (*P* value = 0.19). The mean ± standard deviation of the age at the low pro-BNP level was 30.88 ± 6.23, and the high pro-BNP level was 31.26 ± 4.74 (*P* value = 0.71 and test value =—0.36).

We investigated the cardiac injury biomarker pro-BNP levels in the patient's serum. 49.1% of patients had pro-BNP levels above the cut-off value for predicting an adverse outcome of COVID-19 (Table 1). The mean ± standard deviation of pro-BNP levels in low group was 46.12 ± 17.52 pg/mL and in high group was 878.81 ± 1038.06 pg/mL.

**Table 1:** General characteristics of included pregnant patients according to the level of pro-BNP

Variables		Pro-BNP levels			Test Value	*P*-value
		86 > n (%)	≥ 86 n (%)	Total n (%)		
Vaccination	No	42 (49.4)	43 (50.6)	85 (100)	0.33	0.56
	Yes	14 (56)	11 (44)	25 (100)		
MI	No	56 (53.3)	49 (46.7)	105 (100)	5.43	0.02
	Yes	0 (0)	5 (100)	5 (100)		
Arrhythmia	No	56 (52.8)	50 (47.2)	106 (100)	4.30	0.05
	Yes	0 (0)	4 (100)	4 (100)		
Heart block	No	56 (51.4)	53 (48.6)	109 (100)	1.04	0.49
	Yes	0 (0)	1 (100)	1 (100)		
PE	No	56 (54.4)	47 (45.6)	103 (100)	7.75	< 0.01
	Yes	0 (0)	7 (100)	7 (100)		
Valves diseases	No	45 (52.9)	40 (47.1)	85 (100)	0.37	0.53
	Yes	12 (48)	13 (52)	25 (100)		
Urgent C/S	No	55 (55)	45 (45)	100 (100)	7.36	< 0.01
	Yes	1 (10)	9 (90)	10 (100)		
Mortality	No	56 (53.3)	49 (46.7)	105 (100)	5.43	0.02
	Yes	0 (0)	5 (100)	5 (100)		
Gravid	G1	18 (62.1)	11 (37.9)	29 (100)	5.23	0.14
	G2	18 (51.4)	17 (48.6)	35 (100)		
	G3	12 (57.1)	9 (42.9)	21 (100)		
	G ≥ 4	8 (32)	17 (68)	25 (100)		
Trimester	First	18 (62.1)	11 (37.9)	29 (100)	3.75	0.15
	Second	25 (42.4)	34 (57.6)	59 (100)		
	Third	13 (59.1)	9 (40.9)	22(100)		

*MI* Myocardial infarction, *PE* Pericardial effusion, *C/S* Caesarean Section. *P*-value less than 0.05 considered statistically significant

Most of the patients were pregnant for the first and second times. 4.5% of patients were in the first trimester, 17.8% in the second, and 78.2% in the third. There was no significant relation between gravid and trimester with pro-BNP levels (*P* value = 0.14 and *P*-value = 0.19).

Twenty-five patients (22.7%) had a history of vaccination against COVID-19, and 77.3% had no history of vaccination. There is no significant relation between injection or non-injection of vaccine and pro-BNP level (*P*-value = 0.56 and Test value = 0.33).

Five patients (4.5%) had an acute ST elevation MI on admission, as evidenced by an electrocardiogram. All five patients had pro-BNP levels above the cut-off value, and the results indicate a significant relation between MI and high pro-BNP levels (*P*-value = 0.02 and Test value = 5.43).

Our study indicated no significant relation between arrhythmia, heart block, and valve diseases by pro-BNP level (*P*-value = 0.05, *P*-value = 0.49, and *P*-value = 0.53) by using Fisher's exact test.

At Echo assessment, 95.5% had normal EF, and seven patients (6.4%) had pericardial effusion, and there was a significant relation between PE and pro-BNP level (*P* value =  < 0.01, test value = 7.75).

Ten patients were admitted for emergency caesarean section (C/S); unfortunately, five died. There was a significant relation between urgent C/S and mortality with pro-BNP levels (*P* value =  < 0.01 and *P*-value = 0.02). The risk of emergency C/S was 11 times higher in those with high pro-BNP than those with low pro-BNP levels.

## Discussion

There is insufficient research on the management and adverse effects of COVID-19 in different trimesters of pregnancy. Ideal pregnancy management during the COVID-19 pandemic is critical because pregnant women are at higher risk for clinical course, increased rates of pregnancy complications, medication efficacy, the optimal delivery route, the safety of breastfeeding, and the risk of vertical transmission [11].

Our study revealed that patients with higher pro-BNP plasma levels tended to be at higher risk of adverse clinical features. We investigated cardiovascular complications such as MI, arrhythmia, block, PE, and valve diseases. The results reported no significant relation between gravid, trimester, vaccination, arrhythmia, heart block, and valves diseases with high pro-BNP level. However, the evidence of the study showed a significant relation between MI, PE, urgent C/S, and mortality with high pro-BNP levels.

Cardiac injury is a common complication in hospitalized patients with COVID-19. The exact contribution of COVID-19's cardiac injury is still unclear. However, some studies have reported that inflammation and oxidative stress caused by cytokine storms induce coagulopathy and microangiopathy, leading to perfusion defects and myocardial injury. Additionally, infections can lead to an imbalance between high oxygen demand and low oxygen supply, leading to increased ventricular wall stress and the release of pro-BNP. A study by Huang C., et al. 2020, mentioned that 12% of patients had an acute cardiac injury, and those admitted to the intensive care unit had more developed heart injuries (31%) than those in the non-intensive care unit (4%) [5, 7, 8, 15–20].

Pregnancy, due to physiological changes in the immune and cardiopulmonary systems, can increase cardiac output, plasma volume, diaphragmatic elevation, oxygen consumption, airway mucosa edema, and heart rate. In addition, the signs and symptoms of pregnancy and COVID-19 infection can mimic the symptoms of decompensated heart disease, so the clinical diagnosis of cardiac disease in these cases is difficult. The use of serum pro-BNP levels for rapid diagnosis of cardiac disease in pregnant patients with COVID-19 could be essential. During pregnancy, elevated BNP levels are physiological due to volume overload and ventricular dilatation, with mean values ranging from 15.5 to 19 pg/mL versus a mean of 10 pg/mL. mL in non-pregnant patients has been reported. The study by Sheikh M., et al.2021, states that a BNP level at a cut-off value of 100 pg/mL has a sensitivity of 90% and an accuracy of 83% for pregnant patients and has the same level of sensitivity and accuracy for the non-pregnant population. However, baseline BNP levels were higher in pregnant and postpartum women [9, 12, 13, 20–25]. In the study of Chehrazi M.,et al.2022, who researched 272 patients with COVID-19 without a history of heart disease, they found that a high level of pro-BNP at the time of hospitalization is a predictive factor for the severity of COVID-19 and its mortality. In this study, the consecutive levels of pro-BNP during the hospitalization of the patients were also investigated. They found that higher pro-BNP levels at admission were associated with poor prognosis, and these patients had higher levels during hospitalization. Therefore, a high level of this marker at admission can play a role in predicting the outcome of COVID-19 in patients [10].

Pro-BNP is a neurohormone secreted or increased in response to raised myocardial wall stress, acute kidney injury, and systematic inflammation. Our study found that Severe COVID-19 patients with high pro-BNP levels tended to have increased cardiac injury markers, elevated systematic inflammation markers, and a low survival rate. The high pro-BNP levels in pregnant women could independently predict urgent C/S and mortality. The study by Gao L.,et al.2020, showed that the best pro-BNP cut-off value for predicting mortality was 88.64 pg/mL with 100% sensitivity and 66.67% specificity. Patients with high pro-BNP values (> 88.64 pg/mL) had a significantly increased risk of death during follow-up days compared with patients with low values (≤ 88.64 pg/mL). A study by Fernandez A.,et al.2021, states that pro-BNP levels at admission are independent and complementary predictors of COVID-19 severity and mortality [7, 15, 18, 23–28].

Most patients (77.3%) did not have history of vaccination and there was no significant relation between injection of vaccine with high pro-BNP level. Most of our cases were infected at the beginning of the COVID-19 pandemic, and approved vaccination of pregnant women had not been discovered. In addition, pregnant women's fear of the vaccine's uncertain effects on them and their fetuses' health had led to a decrease in the vaccination rate of pregnant women. This may be the reason for the lack of a statistically significant correlation between vaccination and pro-BNP levels.

Cardiac arrhythmias are seen in viral infection, including atrial fibrillation, conduction block, ventricular tachycardia, and ventricular fibrillation. Arrhythmias result from metabolic dysfunction, myocardial inflammation, and sympathetic nervous system activation during infection. In this study, arrhythmia has a high rate of abnormal ECG changes related to high pro-BNP levels, possibly due to lung injury due to the extensive invasion of COVID-19 or cardiac insufficiency. The study by Alhogbani T., et al.2016, found that 31% of patients infected with COVID-19 had complications such as arrhythmia (such as atrial fibrillation) and cardiac block [4, 7, 8, 19, 25, 29–34].

We found that all abnormal ST-T segment changes were the most common ECG presentation in COVID-19 patients. MI could be related to hypocalcemia, coronary artery disease, hypertension, or myocardial damage caused by COVID-19 infection. This study showed that elevated pro-BNP levels were the independent factors of MI in ECG. The study by Chaolin H.,et al.2020, suggested that COVID-19 infection causes myocardial infarction and increases markers of myocardial damage with clinical evidence of MI on electrocardiogram [16, 17, 23, 25, 30–37].

Pericardial effusion in a patient with COVID-19 has resulted from a severe inflammatory process associated with underlying myocarditis or pericarditis. In this study, using an echocardiographic examination, PE had a static relation with a high pro-BNP level. In patients with elevated pro-BNP, echocardiography can detect pericardial effusion in patients hospitalized for COVID-19 infection. A study by Ghantous E.,et al.2022, reported that 14%% of patients had a high prevalence of PE, but the severity of pericardial effusion was mild. Also, increased values of cardiac enzymes (such as pro-BNP) were associated with pericardial effusion [7, 21, 28, 34, 38–41].

This study reported a significant relation between urgent C/S and mortality with high pro-BNP levels. The reasons for urgent C/S and mortality in these patients could be ischemic and inflammatory processes of COVID-19, reduction of health care, and reduction of maternity services. The study by Mascio D.,et al.2020, mentioned that among hospitalized pregnant patients with COVID-19 infection, the most common adverse pregnancy outcomes were caesarean delivery, preterm birth, and maternal mortality compared with before the pandemic [5, 6, 16, 20, 40–45].

In our study, during the 18 months of the COVID-19 pandemic, five pregnant patients died from this infection, which had a significant relation with elevated pro-BNP levels. The study by Kumari V.,et al.2020, observed a significantly increased in-hospital mortality among pregnant women (0·20 vs. 0·13%; *p* = 0·01) during the post-lockdown period, compared with the pre-lockdown period [6, 46–50].

This study has some limitations and strengths. This study has investigated the role of the pro-BNP factor as a diagnostic tool for cardiovascular complications in pregnant women, which few studies have evaluated this matter. Moreover, we have used echocardiography and EKG diagnostic techniques to confirm heart complications. Our study was retrospective, and some specific information was incomplete due to the limited circumstances of the lockdown and the urgency of containing the COVID-19 pandemic. Also, extensive cohort studies are needed to validate our conclusions because of limited sample size and a single test of NT-proBNP at admission. In most cases, the cause of mortality may be related to multiple organ failure, and it is not easy to distinguish whether the patient's leading direct cause is cardiovascular complications.

## Conclusion

Our research reported that patients with higher pro-BNP levels had a significant relation between MI, PE, urgent C/S, and mortality. Further, no significant relation between gravid, trimester, vaccination, arrhythmia, heart block, and valves diseases with high pro-BNP level was seen. Pro-BNP levels of pregnant COVID-19 patients on admission are useful in early identification of patients with poor prognosis, and this marker is an independent risk factor for cardiovascular complications and mortality.

## Data Availability

The data supporting this study's findings are available from the corresponding author upon reasonable request.

## References

[1] WHO. Coronavirus disease (COVID-19) pandemic. Cited 2021 March 19. Available from: https://www.who.int/emergencies/diseases/novel-coronavirus-2019 .

[2] CDC. 2019 Novel Coronavirus, Wuhan, China. CDC. Cited 2021 March 19. Available from: https://www.cdc.gov/coronavirus/2019-ncov/about/index.html.

[3] Lashkarbolouk N, Mazandarani M, Pourghazi F, Eslami M, MohammadianKhonsari N, Nouri Ghonbalani Z, Ejtahed HS, Qorbani M (2022). How did lockdown and social distancing policies change the eating habits of diabetic patients during the COVID-19 pandemic? A systematic review. Front Psychol.

[4] Wang Y, Chen L, Wang J, He X, Huang F, Chen J, Yang X (2020). Electrocardiogram analysis of patients with different types of COVID-19. Ann Noninvasive Electrocardiol.

[5] Di Mascio D, Khalil A, Saccone G, Rizzo G, Buca D, Liberati M, Vecchiet J, Nappi L, Scambia G, Berghella V, D’Antonio F (2020). Outcome of coronavirus spectrum infections (SARS, MERS, COVID-19) during pregnancy: a systematic review and meta-analysis. Am J Obstet Gynecol MFM.

[6] Kumari V, Mehta K, Choudhary R (2020). COVID-19 outbreak and decreased hospitalisation of pregnant women in labour. Lancet Glob Health.

[7] Gao L, Jiang D, Wen XS, Cheng XC, Sun M, He B, You LN, Lei P, Tan XW, Qin S, Cai GQ (2020). Prognostic value of NT-proBNP in patients with severe COVID-19. Respir Res.

[8] Chmielewska B, Barratt I, Townsend R, Kalafat E, van der Meulen J, Gurol-Urganci I, O'Brien P, Morris E, Draycott T, Thangaratinam S, Le Doare K (2021). Effects of the COVID-19 pandemic on maternal and perinatal outcomes: a systematic review and meta-analysis. Lancet Glob Health.

[9] Sheikh M, Ostadrahimi P, Salarzaei M, Parooie F (2021). Cardiac complications in pregnancy: a systematic review and meta-analysis of diagnostic accuracy of BNP and N-terminal pro-BNP. Cardiol Ther.

[10] Chehrazi M, Yavarpour H, Jalali F, Saravi M, Jafaripour I, Hedayati MT, Amin K, Pourkia R, Abroutan S, Javanian M, Ebrahimpour S (2022). Optimal cut points of N-terminal of the prohormone brain natriuretic peptide (NT-proBNP) in patients with COVID-19. Egypt Heart J.

[11] Karaşin SS, Bayram F (2023). Impact of COVID-19 disease on obstetric outcomes in the third trimester of pregnancy. Eur Res J.

[12] Lazar M, Barbu EC, Chitu CE, Anghel AM, Niculae CM, Manea ED, Damalan AC, Bel AA, Patrascu RE, Hristea A, Ion DA (2022). Pericardial Involvement in Severe COVID-19 Patients. Medicina.

[13] Mayama M, Yoshihara M, Uno K (2017). Factors influencing brain natriuretic peptide levels in healthy pregnant women. Int J Cardiol.

[14] Sharififar R, Heidari K, Mazandarani M, Lashkarbolouk N. Comparison of the Polymerase Chain Reaction Method with Serological Tests in the Diagnosis of Human Brucellosis. Jundishapur J Microbiol. 2023;16(2).

[15] Jayakrishnan MP, Sindhu TG, Sadik KC, Ajithkumar VT, Rajesh GN (2022). Electrocardiographic Abnormalities in Multisystem Inflammatory Syndrome Related to COVID-19. Indian J Pediatr.

[16] Naeem A, Tabassum S, Gill S, Khan MZ, Mumtaz N, Qaiser Q (2023). COVID-19 and cardiovascular diseases: a literature review from pathogenesis to diagnosis. Cureus.

[17] Huang C, Wang Y, Li X (2020). Clinical features of patients infected with 2019 novel coronavirus in Wuhan China. Lancet.

[18] Lalani K, Seshadri S, Samanth J, Thomas JJ, Rao MS, Kotian N, Satheesh J, Nayak K (2022). Cardiovascular complications and predictors of mortality in hospitalized patients with COVID-19: a cross-sectional study from the Indian subcontinent. Trop Med Health.

[19] Fremed MA, Healy EW, Choi NH, Cheung EW, Choudhury TA, Jiang P, Liberman L, Zucker J, Lytrivi ID, Starc TJ (2022). Elevated cardiac biomarkers and outcomes in children and adolescents with acute COVID-19. Cardiol Young.

[20] Liu A, Hammond R, Donnelly PD, Kaski JC, Coates AR (2023). Effective prognostic and clinical risk stratification in COVID-19 using multimodality biomarkers. J Internal Med.

[21] Maddury J, Krishna M, Kumar A (2022). Cardiovascular manifestations in COVID-19 patients. Apollo Medicine.

[22] Battaglini D, Lopes-Pacheco M, Castro-Faria-Neto HC, Pelosi P, Rocco PR (2022). Laboratory biomarkers for diagnosis and prognosis in COVID-19. Front Immunol.

[23] Sauer F, Dagrenat C, Couppie P, Jochum G, Leddet P (2020). Pericardial effusion in patients with COVID-19: case series. Eur Heart J.

[24] Goel S, Monica MB, Garg V, Aggarwal PK, Mittal A, Agarwal A, Garg AK (2022). ECG abnormalities in patients affected with COVID-19: a review. J Pharm Negative Results.

[25] Shafi AM, Shaikh SA, Shirke MM, Iddawela S, Harky A (2020). Cardiac manifestations in COVID-19 patients—A systematic review. J Card Surg.

[26] Lopes V, Baptista JP, Moreira N, Goncalves L (2022). Admission NT-proBNP and outcomes in critically ill COVID-19 patients. Eur Heart J.

[27] Cersosimo A, Cimino G, Amore L, Calvi E, Pascariello G, Inciardi RM (2023). Cardiac biomarkers and mortality in COVID-19 infection: a review. Monaldi Arch Chest Dis.

[28] Orlando L, Bagnato G, Ioppolo C, Franzè MS, Perticone M, Versace AG (2023). Natural course of COVID-19 and independent predictors of mortality. Biomedicines.

[29] Alhogbani T (2016). Acute myocarditis associated with novel Middle East respiratory syndrome coronavirus. Ann Saudi Med.

[30] Asif M, Waqas M, Razzaq H, Choudhry SJ, Arshad N, Nazeer N (2022). NT PROBNP'S prognostic value among patients of Covid-19 without heart failure. Pakistan J Med Health Sci.

[31] Chaolin H, Yeming W, Xingwang L, Lili R, Jianping Z, Yi H (2020). Clinical features of patients infected with 2019 novel coronavirus in Wuhan, China. Lancet.

[32] Nathala P, Salunkhe V, Samanapally H, Xu Q, Furmanek S, Fahmy OH, Deepti F, Glynn A, McGuffin T, Goldsmith DC, Petrey J (2022). Electrocardiographic features and outcome: Correlations in 124 hospitalized patients with COVID-19 and cardiovascular events. J Cardiothorac Vasc Anesth.

[33] Mahase ECOVID-19 (2020). WHO declares pandemic because of “alarming levels” of spread, severity and inaction. BMJ..

[34] Ghantous E, Szekely Y, Lichter Y, Levi E, Taieb P, Banai A, Sapir O, Granot Y, Lupu L, Hochstadt A, Merdler I (2022). Pericardial involvement in patients hospitalized with COVID-19: prevalence, associates, and clinical implications. J Am Heart Assoc.

[35] Calvo-Fernandez A, Izquierdo A, Subirana I, Farre N, Vila J, Duran X, Garcia-Guimaraes M, Valdivielso S, Cabero P, Soler C, Garcia-Ribas C (2021). Markers of myocardial injury in the prediction of short-term COVID-19 prognosis. Revista Española de Cardiología (English Edition).

[36] Zou L, Ruan F, Huang M (2020). SARS-CoV-2 viral load in upper respiratory specimens of infected patients. N Engl J Med.

[37] Singhal TA (2020). Review of Coronavirus Disease-2019 (COVID-19). Indian J Pediatr.

[38] Michelen M, Jones N, Stavropoulou C In patients of COVID-19, what are the symptoms and clinical features of mild and moderate cases. Cited 2021 March 19. Available from: https://www.cebm.net/covid-19/in-patients-of-covid-19-what-are-the-symptoms-andclinical-features-of-mild-and-moderate-case .

[39] Yang X, Yu Y, Xu J (2020). Clinical course and outcomes of critically ill patients with SARS-CoV-2 pneumonia in Wuhan, China: a single-centered, retrospective, observational study. Lancet Respir Med.

[40] Zhou F, Yu T, Du R (2020). Clinical course and risk factors for mortality of adult inpatients with COVID-19 in Wuhan, China: a retrospective cohort study. Lancet.

[41] Warning JC, McCracken SA, Morris JM (2011). A balancing act: mechanisms by which the fetus avoids rejection by the maternal immune system. Reproduction.

[42] Zambrano LD, Ellington S, Strid P, Galang RR, Oduyebo T, Tong VT (2020). Update: characteristics of symptomatic women of reproductive age with laboratory-confirmed SARS-CoV-2 infection by pregnancy status – United States, January 22– October 3, 2020. MMWR (Morb Mortal Wkly Rep).

[43] Haitao T, Vermunt JV, Abeykoon J, Ghamrawi R, Gunaratne M, Jayachandran M (2020). COVID-19 and sex differences: mechanisms and biomarkers. Mayo Clin Proc.

[44] Sánchez BG, Gasalla JM, Sanchez-Chapado M, Bort A, Diaz-Laviada I. Potential use of pregnancy-associated plasma protein-A and IMA as biomarkers for the early stage of COVID-19. In press. 10.3390/jcm10235474 PMC8658290 34884175

[45] Guzik TJ, Mohiddin SA, Dimarco A, Patel V, Savvatis K, Marelli-Berg FM, Madhur MS, Tomaszewski M, Maffia P, D’acquisto F, Nicklin SA (2020). COVID-19 and the cardiovascular system: implications for risk assessment, diagnosis, and treatment options. Cardiovasc Res.

[46] Yan-Rong G, Qing-Dong C, Zhong-Si H (2020). The origin, transmission and clinical therapies on coronavirus disease 2019 (COVID-19) outbreak-an update on the status. Mil Med Res.

[47] Antia R, Halloran ME (2021). Transition to endemicity: understanding COVID-19. Immunity.

[48] Klocek M, Wojciechowska W, Terlecki M, Pavlinec C, Grodzicki T, Małecki M, et al. Cardiac biomarkers on admission and in-hospital mortality in COVID-19 patients with or without concomitant heart failure. Polish Arch Internal Med. 2022;132(7–8). 10.20452/pamw.16256 35522239

[49] Sano M, Toyota T, Morimoto T, Okada T, Sasaki Y, Taniguchi T, Kim K, Kobori A, Ehara N, Kinoshita M, Doi A (2022). Prediction of clinical outcomes in patients with coronavirus disease 2019 using high-sensitive troponin I and N-terminal pro-B-type natriuretic peptide. Eur Heart J.

[50] Pranata R, Huang I, Lukito AA, Raharjo SB (2020). Elevated N-terminal pro-brain natriuretic peptide is associated with increased mortality in patients with COVID-19: systematic review and meta-analysis. Postgrad Med J.

